# Pathogen recognition by sensory neurons: hypotheses on the specificity of sensory neuron signaling

**DOI:** 10.3389/fimmu.2023.1184000

**Published:** 2023-05-03

**Authors:** Antoine Millet, Nicholas Jendzjowsky

**Affiliations:** ^1^ Respiratory & Exercise Physiology, The Lundquist Institute for Biomedical Innovation at Harbor University of California Los Angeles (UCLA) Medical Center, Torrance, CA, United States; ^2^ Division of Respiratory and Critical Care Medicine and Physiology, David Geffen School of Medicine, University of California Los Angeles (UCLA), Los Angeles, CA, United States

**Keywords:** pathogen recognition receptors (PRR), pathogen associated molecular patterns (PAMPs), transient receptor potential channel, sensory neuron, dorsal root ganglion (DRG), vagus, nodose ganglion (NG), carotid chemoreceptors

## Abstract

Sensory neurons cooperate with barrier tissues and resident immune cells to form a significant aspect of defensive strategies in concert with the immune system. This assembly of neuroimmune cellular units is exemplified across evolution from early metazoans to mammalian life. As such, sensory neurons possess the capability to detect pathogenic infiltrates at barrier surfaces. This capacity relies on mechanisms that unleash specific cell signaling, trafficking and defensive reflexes. These pathways exploit mechanisms to amplify and enhance the alerting response should pathogenic infiltration seep into other tissue compartments and/or systemic circulation. Here we explore two hypotheses: 1) that sensory neurons’ potential cellular signaling pathways require the interaction of pathogen recognition receptors and ion channels specific to sensory neurons and; 2) mechanisms which amplify these sensing pathways require activation of multiple sensory neuron sites. Where possible, we provide references to other apt reviews which provide the reader more detail on specific aspects of the perspectives provided here.

## Introduction

Without the ability to detect, combat and evade harmful pathogens, the survival of early metazoan and, evolution to mammalian life would not have been possible (1). The importance of pathogenic detection and mitigation is evidenced by innate systems, mostly confined to the earliest sensory units, which originated in early multicellular organisms such as *Caenorhabditis elegans* (2, 3) and Hydra (4, 5). The mammalian development of pathogenic control was enabled by the divergence from one to two systems which evolved in parallel but in a mutually interrelated manner (1, 3). Mammalian immune and nervous systems are organized in such a way that sentinel cells, with pathogenic detecting capabilities emanate alerting signals. When amplified chemical messengers are released to recruit combative cells that attack and/or eliminate invading threats (1).

Layers of defenses are involved in the intricacy of the immune system. Structural barrier cells within epithelial barrier layers coordinate a signalling cascade to recruit additional patrolling and cytotoxic cells, granulocytes, monocytes and lymphocytes to act and counter the invading threat. Following the cellular signalling posed by the epithelium, the immune system is then organized into lymphoid and secondary lymphoid organs, which harbor lymphoid-lineage cells that enable bi-directional communication with myeloid lineage cells, which have multiple methods to neutralize pathogens. Lymphoid lineage cells are triggered by numerous signals, simultaneously deployed at any one time, which was thought initially to be a failsafe system to back-up disruptions to poorly performing pathways (6, 7). However, the diversity and coordination of multiple cytokine and chemokine release appear to mediate the specificity of immune cell activation in response to pathogenic detection. This is due to the unique receptor gene expression profile on any single immune cell, providing a unique signature and the potential for differing modes of activation (8–10). Thus, deployment of a specific cytokine and chemokine pattern would then, presumably, activate a particular set of cells, in an exact way, to target a specific pathogen.

Like the immune system, the nervous system is partitioned to enable the sensing of the environment (sensory neurons within the peripheral nervous system), integrate and process information (medullary autonomic nervous system – and central hypothalamic centers) and finally, carry out signalling pathways (efferent nerves of the autonomic nervous system) to implement reflexes to receive information regarding deviations from homeostasis (1, 3). In the case of immediate pathogenic invasion, sensory neurons innervate all barrier (11–13) and mucosal surfaces (11–13) and patrol the bloodstream (14–16); interfaces which are vulnerable to infection and can come into contact with the outside environment. These important neural sensory clusters lie within the dorsal root ganglion (13), trigeminal ganglion (17), nodose/jugular ganglion (11, 18) and petrosal ganglion (14, 18), respectively. These neurons communicate with immune (19) and neuroendocrine cells (20, 21), either by the direct release of neurotransmitters into the immediate vicinity (19) or by relaying more extensive neural networks to innervate secondary lymphoid organs (22) and recruit other cells via secondary neurotransmitter release.

As such, it is clear that the anatomical association of sensory neurons with epithelial layers and indwelling immune cells form “neuroimmune cell units” (23) where their bidirectional communication between distinct neuronal and hematopoietic lineage cell types has afforded the ability to direct specific responses to a multitude of homeostatic disturbances. Prominent examples of the effects of these neuroimmune cell units are the cholinergic anti-inflammatory pathway (24, 25) or sympathetic influence on lymphopoiesis (26, 27). These modes of neural stimulation of immune function provide a sensory system which can quickly act should it identify pathogenic infiltration. Notably, the stimulation of this fast-acting sensory neuron pathogenic detection has been demonstrated to either assist with ‘immune’ cell recruitment (19) or provide antimicrobial protection in and of itself through antimicrobial neuropeptides (28). If the specific recruitment of immune cell subsets occurs due to the unique chemokine receptor expression signature on any immune cell (5, 6), what is the mode of specific sensory neuron activation in the face of pathogenic detection?

## Early sensory systems are a blueprint for neuroimmune cell units

Pathogen recognition in early metazoan life likely developed in sensory neurons first (3). These sensory units acquired innate defenses to detect and counter initial pathogenic infiltration. The concurrent development of early PRRs and ion channels shows a sharing of potential physiologic outcomes in response to pathogen detection. Both pathogenic opsonization and sensory neuron depolarization induce avoidance behaviors brought on upon specific neurotransmitter release. This subsequent release of neuropeptides also demonstrates microbial neutralization properties akin to bacteriocins (29, 30); which appears to be conserved in mammalian life (28). Neuronal cell death to implement calcium release for both additional cell recruitment and adjacent neuron stimulation (31, 32) was an additional strategy of early alerting responses and, in select cases, is a last resort for mammalian defences (33). Intriguingly, it would appear that apoptotic signalling and not apoptosis per se, are necessary for neuroimmune interactions as blockade of cell death does not diminish immune cell trafficking (34). Nevertheless, these early alerting responses occur rapidly, and it has been suggested that the speed of the neural response may influence the speed of the subsequent immune response (3, 34); a relationship which appears to hold in mammalian life (19, 33). However, early strategies evolved and gained more specificity with the increased dispersion of duties amongst cell types. The conservation of intracellular signalling pathways throughout the evolution of defensive strategies is exemplified in neuroimmune cell units (11–13, 35–39). How these signalling systems interact, and their functional outcome are now the focus of intense investigation.

## Hypotheses on the specificity of sensory neuron PRR sensing of microbes

In the case of sensory neuron depolarization, ion channels often form the terminal portion of the cell signalling cascade as these channels are phosphorylated by upstream kinases set in motion when receptors are activated (40). The phosphorylation of ion channels on sensory neurons then increases the probability of depolarization, leading to neuronal excitation and neurotransmitter release (41, 42). Neuronal ion channel expression has allowed the sorting/clustering of neuronal subsets (11–13, 35–39). It is likely that the complexity of neural depolarization mediated by the activation of multiple but sensory-specific ion channels in various ganglia would also enable a failsafe method to carry important information to the central nervous system (35, 43). It is equally likely that the specificity of neuronal activation also involves the activation of multiple receptors on any one neuron to offer specific neuron excitation. A notion carried from specific lymphoid cell activation afforded by chemokine receptor expression (8–10).

Furthermore, how and at which site of phosphorylation of each channel could offer another layer of complexity to add to the specific coding of neuronal activation by upstream receptors. Finally, subclusters of specific sensory neurons exist in multiple ganglia; can stimulation of neurons in multiple ganglia may augment neuroimmune reflexes? Therefore, the perspectives explored here are: whether the stimulation of multiple neurons within or between sensory ganglia, are phosphorylated differentially, and on differential phosphorylation sites, to provide a mechanism to code specific information to engage the appropriate neuroimmune reflex. In addition, the activation of ion channel phosphorylation on more than one set of nerves (ganglia) could augment these alerting reflexes.

## Evidence for specificity of neuronal sub-clusters and ion channel phosphorylation as a means for specific neural responses

**Figure 1:**
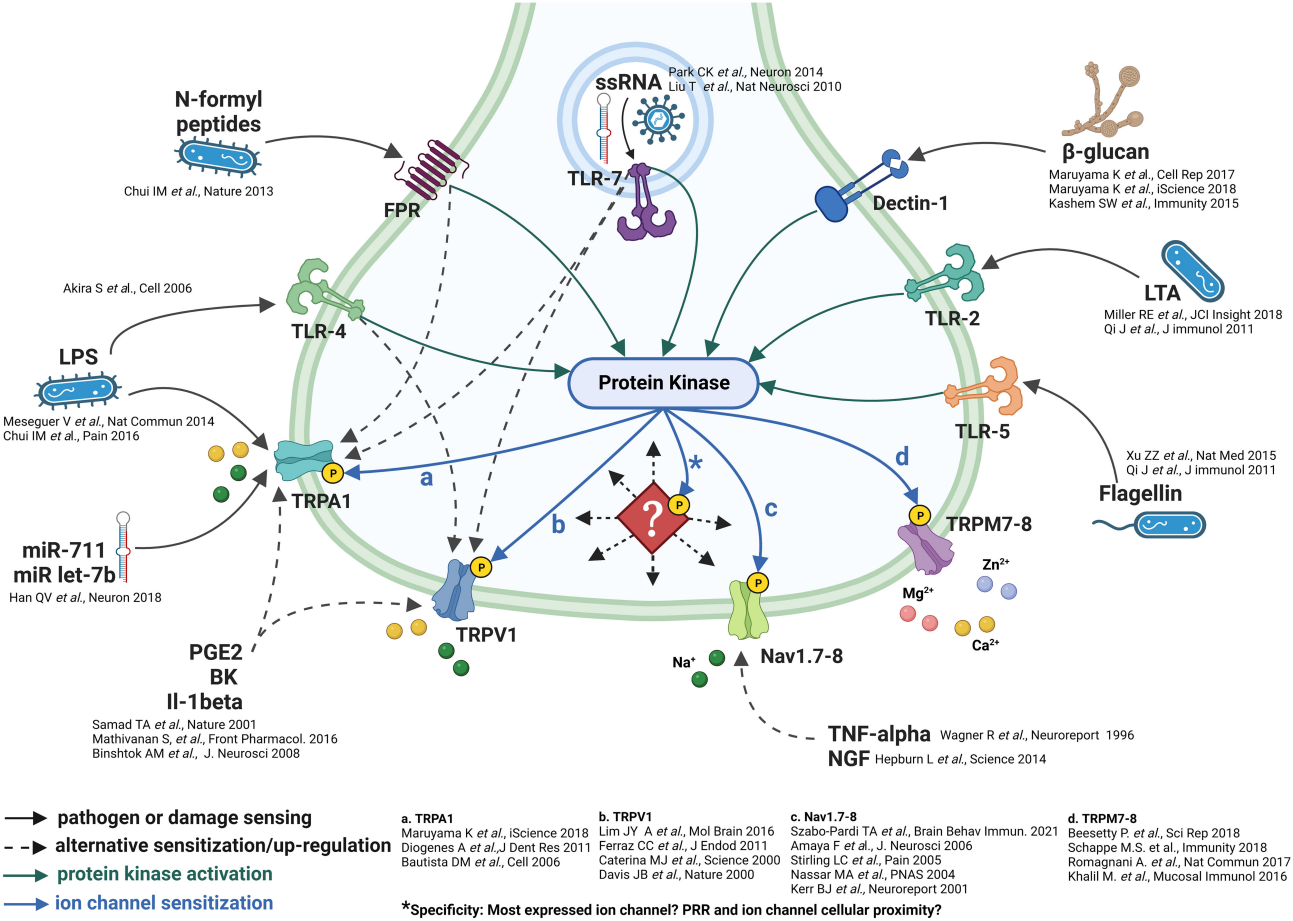
Neuronal pathogenic detection and cellular signaling pathways. Do ion channels incur specificity? The pathogenic sensing capability of sensory neurons is exemplified by the multitude of pathogenic detection receptors (51–79). These receptors appear to be linked spatially and by protein kinase stimulation/phosphorylation of downstream ion channels. *However, as indicated by the diamond, what forces direct the specific trafficking of protein kinases to specific phosphorylation sites on downstream ion channels? And, to which specific ion channel/set of ion channels?

Detection of pathogenic invasion by sensory neurons, epithelial/barrier and innate antigen sensing/presenting immune cells is afforded by direct and indirect means (44). The direct recognition of pathogen-associated molecular patterns (PAMPs) and, therefore, neural activation involves four families of PRRs, including Toll-like receptors (TLRs), NOD-like receptors (NLRs), retinoic acid-inducible gene I (RIG-I)-like receptors (RLRs), formyl peptide receptors (FPRs) and C-lectin receptors (CLRs), each of which show notable differences regarding pathogen recognition, signal transduction, and intracellular downstream pathways (45–51) (**Figure 1**). The indirect activation of sensory neurons by pathogenic stimuli involves cytokines, chemokine, prostaglandin and, phospholipid stimulation of sensory neurons as a result of their release from either myeloid and lymphoid cells or, on neurons themselves (44, 80).

The differential expression of PRRs, cytokine, chemokine, prostaglandin and phospholipid receptors in sensory neurons has been shown in DRGs and trigeminal ganglia, nodose/jugular ganglia and carotid chemoreceptors using a multitude of transcriptomic technologies including single-cell/differential RNA sequencing (sc/dRNA-seq), *in situ* hybridization, immunohistochemistry, and electrophysiology (11–13, 35–39). This potential for neuronal activation by multiple ligands is clearly a distinct possibility, both by direct (sensing of microbes by PRRs) and indirect (sensing of cytokines released by immune cell sensing of microbes and recognition of alarmins which are produced or released by damaged and dying cells) means. Is it possible that the composition of these molecules released upon initial immune cell sensing and the direct pathogenic stimulation of neurons can code specific neuronal signalling? To test this hypothesis would require a multitude of conditions with differing amounts and compositions of ligands in response to different pathogenic conditions; a difficult task which may be made easier with new-generation bioinformatic approaches involving RNA-protein interactions or the functional processing of neural activity. However, this same stimulation would also activate many other cell types with a similar receptor signature. Therefore, an additional layer of neuronal specificity is required to allow sensory neuron signaling specificity.

The composition of downstream ion channels is the defining characteristic of sensory neurons (11–13, 41). Protein phosphorylation is the most important post-translational protein modification which regulates enzymatic and ion channel activity, cell signalling and cellular localization (40, 81–83). Given the prominent role of protein kinases, which are readily activated as part of multiple signaling pathways (84–86), it is likely that their deployment can elicit downstream post-translational modification of ion channels to increase the probability of depolarization and/or neurotransmitter release which, in turn, could code specific nociceptor signals (**Figure 1**).

There are multiple examples of PRR receptors eliciting the engagement of kinases, including protein kinases, tyrosine, and Syk-family kinases as part of both PRR signalling (84–87) and indirect signaling from phospholipid, prostaglandin and cytokine receptors (87, 88). Further, the role phosphorylation plays in the modification of ion channel function in regards to neuronal depolarization has been documented in response, mainly, to GPCR mediated kinase activation such as prostaglandins and phospholipids (89, 90), and in response to some, but not all, neuroimmune conditions, examples include itch (91) and pain (92, 93). Subsequent activation of kinases in association with PRRs such as TLRs or CLRs and cytokines are certainly evidenced (87); whether their kinase activation results in ion channel depolarization or sensitization in these cases is not clear (**Figure 1**).

An additional possibility, in terms of the specificity of neuronal activation, appears to involve multiple phosphorylation sites per ion channel. The most prominent examples of these, within sensory neurons, lie with TRPV1, TRPC3 and Nav channels. Indeed, TRPV1 has three prominent phosphorylation sites, S502, S800 and T704 (94, 95), TRPC3 is phosphorylated on T11, S263, T646 and (96–98), and sensory neuron specific Nav channels, subtypes 1.8 and 1.9 are phosphorylated on D1-DII linker sites (99, 100). Each of these sites accepts kinases that appear to be directed in response to specific ligands such as prostaglandins and cytokines (40). This array of cellular signaling may be a manner in which multiple ion channels may be simultaneously phosphorylated and afford specificity similar to the multiple chemokine and cytokine stimulation of immune cells (44). The wide-ranging consequences of the multiplicity of signals contributing to ion channel phosphorylation could result in either pro- or anti-inflammatory responses. But an essential factor, which remains unanswered, is how the activation of kinases by specific receptors are directed/trafficked to particular phosphorylation sites on specific ion channels. There are examples of apparent coupling between PRRs and TRPs, such as TLR4 to TRPV1 and TLR7 to TRPA1 (44). What is a more probable cellular signalling scenario is that the dispersion of kinases, in response to stimulation, by a multitude of receptors, phosphorylates/post-translationally modifies the composition of channels present within each specific neuron subset. This would speak to the mode of specificity mentioned above. However, this hypothesis remains to be tested.

## Evidence for multiple ganglion stimulation and alteration of defensive reflexes

In early eukaryotic life, the capability of neural signals to recruit cells from seemingly ‘distant’ sites (3, 101) demonstrated the capability for early neuroimmune cell unit coordination and broader protective capabilities. However, as eukaryotic life gained the complexity and partitioning of organ systems, both the immune and nervous systems organized themselves by sub-dividing specific hubs for either the storage of cells (e.g. secondary lymphoid organs, resident tissue cells) or to serve as a relay station for information (e.g. neural ganglia).

In the case of the sensory nervous system, necessary to form a part of the neuroimmune landscape, neurons are sorted and partitioned into different peripheral ganglia which innervate different organ systems or components of organ systems (19, 102). For example, the dorsal root ganglia innervate the skin (13, 102). In contrast, the vagus senses internal organs, and each neuron/neuron subset is responsible for a single organ system (11, 35, 102) and, possibly, tissue layer (11, 103, 104).

Signals from sensory neurons travel to the nucleus tractus solitarius and synapse onto the dorsal motor nucleus of the vagus (to elicit immediate efferent nerve activity) and the paraventricular nucleus within the hippocampus, amygdala and periaqueductal grey (to carry out grander neuroendocrine signalling involving the hypothalamic pituitary adrenal axis; HPA). Effective neural signalling of immunity is critically dependent on adrenal cortex release of glucocorticoids in response to a number of perturbations (105, 106) The integration of the neuroendocrine involvement in neuroimmune signalling is highlighted by stress responses (involved in a wide variety of threats to homeostasis) which signals the adrenal cortex to induce corticosteroid release which has a wide array of immune influence (105–107). Additionally, new and important reflexes of HPA involvement are highlighted with the cholinergic anti-inflammatory pathway (108) and humoral immunity (109) in response to infection.

Intriguingly, disease and its severity can often be magnified when it is ‘leaked’ beyond the initial site of infection into other tissue layers, organ systems and/or the blood; examples of these lie with infections that can quickly precipitate sepsis (16). Therefore, a situation may arise where multiple neurons that may signal differing ganglia could signal an alerting response should the disease spread beyond the initial site/tissue of infection.

**Figure 2:**
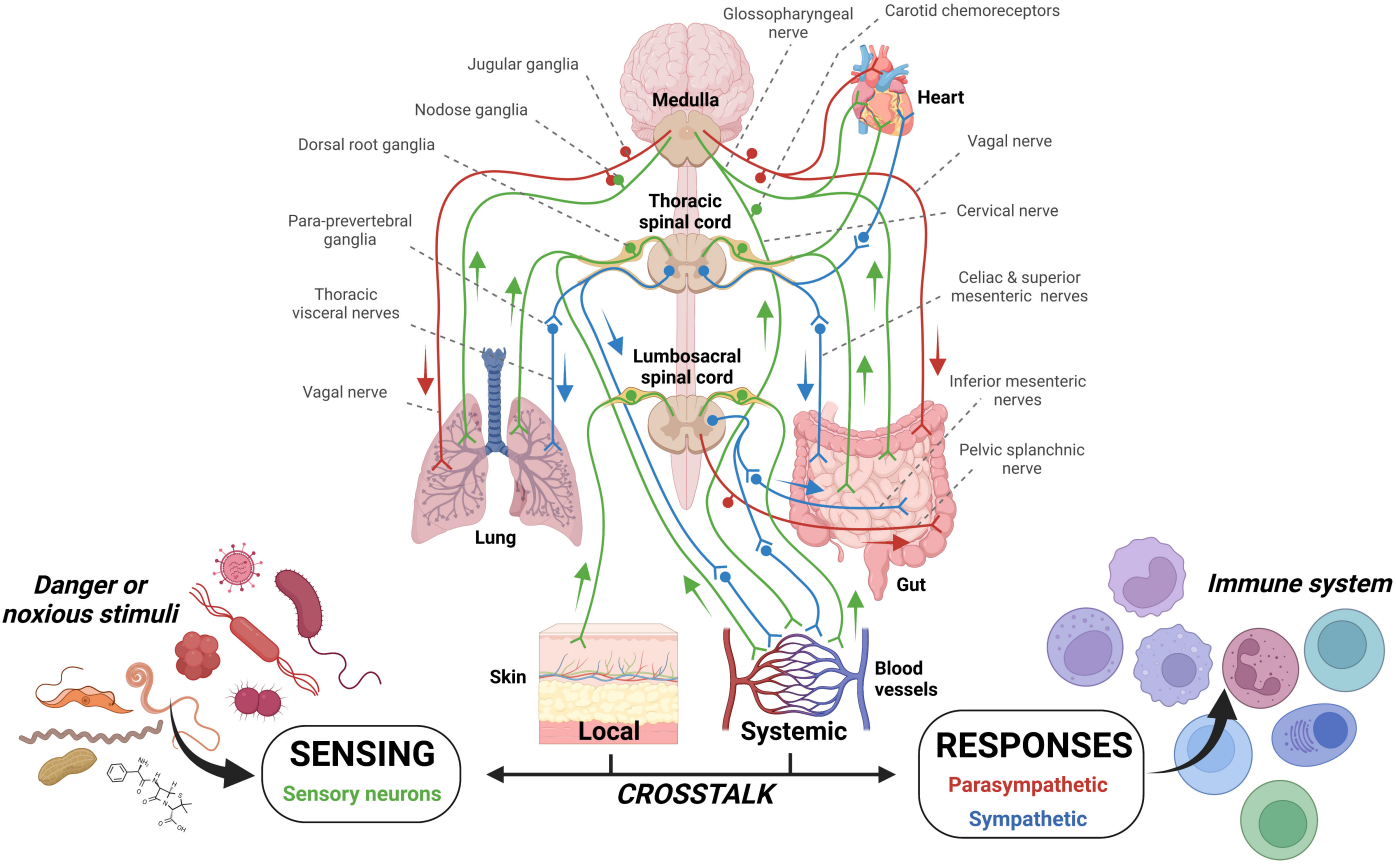
Sensory innervation of barrier tissues. The innervation of tissues by sensory neurons and the parasympathetic and sympathetic arms of the autonomic nervous system affords constant patrol of mammalian systems. In addition, the enteric nervous system has a layered system of innervation within the gut. These systems allow constant detection and also the possibility of dual stimulation should multiple neurons in different locations/tissues be stimulation. Can parallel and concurrent sensory neuron pathogenic stimulation increase signaling magnitude? The potential for this case has been examined previously (43, 103).

One such case where infection could travel beyond the initial site of infection but remain within a single organ system, yet stimulate multiple sensory neurons which are housed in differing ganglia, is exemplified in the gut (103). Indeed, the colon is partitioned into intrinsic and extrinsic sensory neurons, with cell bodies in the intrinsic and nodose ganglia, respectively (103) (**Figure 2**). It is likely that stimulation of both ganglia by the detection of pathogenic harm by both sets of sensory neurons may provide an increased alerting response.

Indeed, the above discussion readily acknowledges that the first line of neuroimmune defence occurs with the release of these sensory neurotransmitters which act to recruit the appropriate cell to clear the pathogenic invasion. However, should disease progress to such a state where barrier surfaces become leaky/decoupled and allow the passage of microbial pathogens and/or cytokines into the bloodstream, additional defenses would be stimulated. In this regard, an additional line of defense would be recruited if pathogenic infiltration should enter the blood stream; the carotid chemoreceptors (16) the main sensory organ which patrols hemolytic homeostasis (15) would also be stimulated. Evidence demonstrates the carotid chemoreceptors’ ability to detect pathogenic patterns (16) as well as substances released in response to disease states/allergy (110, 111) as their composition of pathogenic detection is notable (38). We have demonstrated the possibility for this biologic scenario to occur in our dual vagus and carotid chemoreceptor preparation (43). Specifically, we showed that when the sensory neurons within the lungs are stimulated with either proteases or adenosine triphosphate and the carotid chemoreceptors with their known stimulus, low oxygen tension of their perfusate (blood), signals emanating to the efferent vagus are augmented, likely to regulate defensive reflexes such as mucus secretion and airway constriction (35) and/or to signal the cholinergic anti-inflammatory pathway (24, 25). This scenario shows that not only are subtypes of neurons within ganglia able to regulate and code specific neural signals in response to pathogenic stimulation but, also that augmentation of reflexes is possible when anatomically specific and important ganglia are stimulated at the same time. Therefore, the cascading stimulation of sensory neurons would provide the potential to augment and/or strengthen the neural coordination of immune cells, should pathogenic stimuli be sensed by multiple immune capable ganglia. In the context of specific coding of neural depolarization by the expression of differential receptors, ion channels and their respective phosphorylation, how would stimulation of two neuron sets with similar receptor/ion channel composition augment neuroimmune signalling? One possibility would simply be an increased rate of neuronal firing to elicit immunogenic neurotransmitter release. How would dual stimulation of two neuron sets with differing receptor/ion channel composition augment neuroimmune signalling? Would such an instance change the composition of neurotransmitter release and ultimately affect the chemotaxis of immune cells?

## Perspectives/conclusions

Protection and evasion of disease has required an ever-evolving system with additive layers throughout the evolution of eukaryotic life. With the increasing complexity of mammalian body plans, host defence involves coordinating two defensive systems and organising cell clusters within and between systems. This perspectives essay has attempted to highlight how sensory neurons, an important part of the defensive response to microbial invasion, code specific information to either influence neuronal and immune cell stimulation at the site of infection. Additionally, should invasion extend beyond this site, larger reflexes which may be required to clear the pathogenic infiltration would become activated. However, much remains to be uncovered and, as recently posed by others (44), information regarding what this excitation means in a (patho)physiologic context and how it coordinates the release of neurotransmitter to act directly on immune cells or signal the central nervous system to coordinate grander neuro-immune regulation requires further intense investigation.

## Data availability statement

The original contributions presented in the study are included in the article/supplementary material. Further inquiries can be directed to the corresponding author.

## Author contributions

All authors contributed to the conception and composition of the manuscript. AM completed the figures. All authors approve the manuscript in the submitted form.
